# CO homologation and isocyanide activation by a trisilyl alane radical anion

**DOI:** 10.1039/d6cc02324j

**Published:** 2026-06-08

**Authors:** Yueer Zhu, Xufang Liu, Fiona J. Kiefer, Shigeyoshi Inoue

**Affiliations:** a TUM School of Natural Sciences, Department of Chemistry, Wacker-Institute of Silicon Chemistry and Catalysis Research Center, Technische Universität München Lichtenbergstraße 4 85748 Garching bei München Germany s.inoue@tum.de

## Abstract

We report the divergent reactivities of a trisilyl-substituted alane and its radical anionic species towards isocyanides and carbon monoxide. While the neutral Al(iii) species forms coordination complexes, the Al(ii) radical promotes cyanide formation. Notably, the radical anion mediates CO homologation to yield a C_3_ fragment, which provides new insight into main-group CO homologation.

## Abstract 2

A trisilyl alane radical anion shows divergent reactivity from its Al(iii) precursor, enabling isocyanide C–N bond cleavage and CO homologation to a C_3_ fragment.

## Graphical abstract

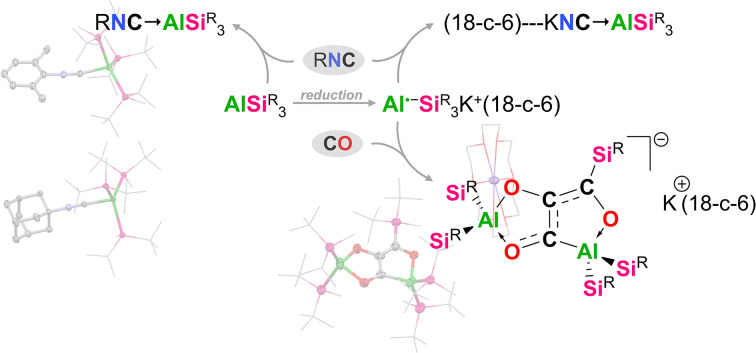

In recent decades, organoaluminium compounds have attracted considerable attention owing to their pronounced reactivity toward small molecules and their key roles in catalysis using earth-abundant elements.^1–6^ Organoalanes containing an Al(iii) center have been extensively studied in this research field. Previously, coordination products of several trialkyl- and triaryl-substituted organoalanes with isocyanides as well as the double insertion product of tri-*tert*-butyl isocyanide into an Al–C bond in 
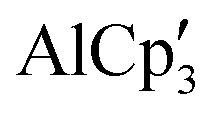
 (Cp′ = C_5_Me_4_H) were isolated and characterized (A–J, Fig. 1a).^7–9^ The carbon monoxide insertion of tri-*tert*-butylalane was also reported (K, Fig. 1a).^10^ In comparison with trialkylalanes, trisilylalanes remain largely unexplored and have attracted considerable research interest due to the steric shielding and electron-donating capabilities of silyl substituents.^11,12^ Since the first trisilylalane, Al(SiMe_3_)_3_, was synthesized in 1980, several alanes with bulkier silyl substituents have been reported.^13–16^ Notably, *via* the reduction of Al(Si^*t*^Bu_2_Me)_3_ (1) with elemental alkali metal, Sekiguchi's group isolated the mononuclear Al(ii) radical anion [Al˙(Si^*t*^Bu_2_Me)_3_]^−^[M]^+^ (2[M], M = K, Na, Li, Fig. 1b).^15^ To the best of our knowledge, no small molecule activation of trisilylalanes has been reported so far. Recent studies have shown that organoaluminium compounds can activate carbon monoxide towards C–O triple bond cleavage and C–C bond coupling.^3,17–21^ This research domain has attracted great research interest as CO is both a key component of the Fischer–Tropsch process and an essential C_1_ building block of many complex molecules.^22,23^ CO homologation of transition metal carbonyl compounds by Al(i) compounds has been reported.^17,19^ Anionic aluminium imide complexes have been shown to be able to incorporate multiple CO molecules, forming C_2_, C_4_ or C_6_ chains (I and II, Fig. 1c).^18,21^ In addition, reduction of CO to a C_4_ chain by an aluminyl anion has been demonstrated (III, Fig. 1c).^20^ More recently, our group reported CO homologation mediated by a neutral alumene (IV, Fig. 1c).^3^ Herein, we report the reactivity of Al(Si^*t*^Bu_2_Me)_3_ (1) and its radical anion 2[K] toward CO and its isoelectronic analogues, isocyanides (Fig. 1d). Various isocyanide complexes (3–6) and a CO homologation product (7) were isolated and characterized.

**Fig. 1:**
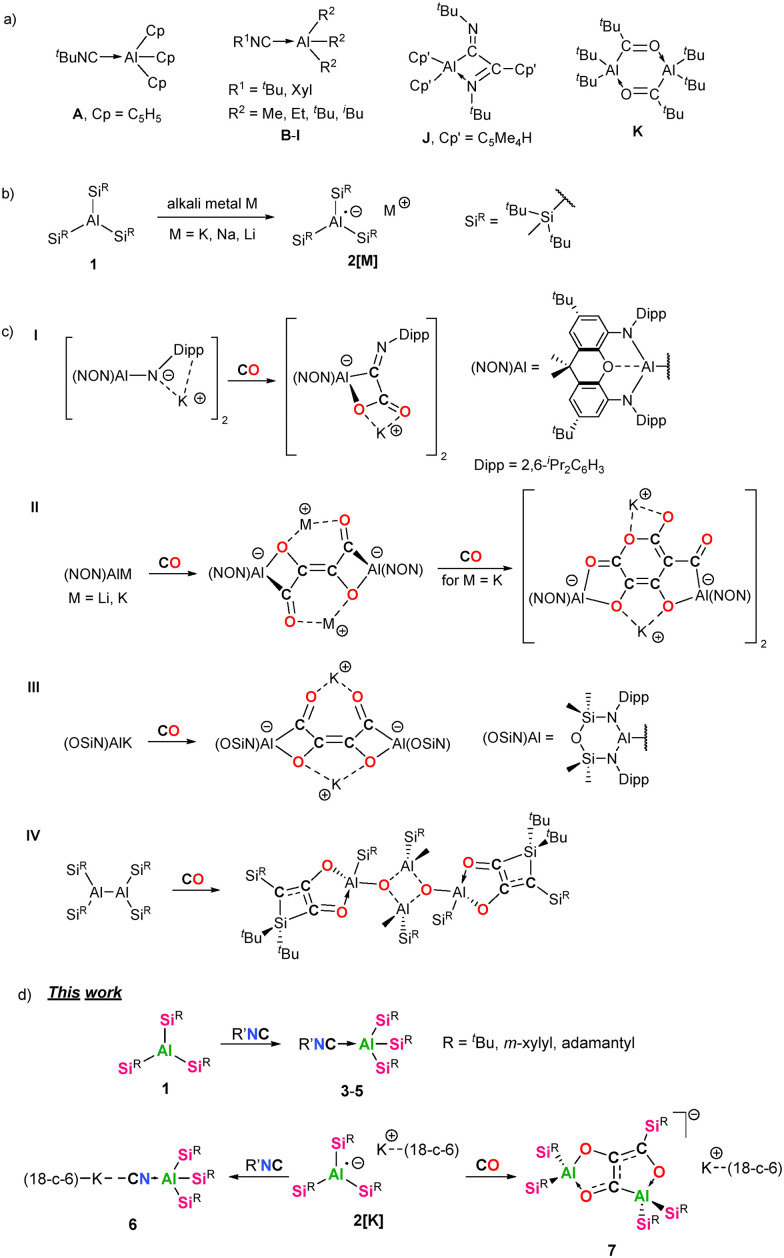
(a) The isocyanide and CO complexes of alanes. (b) The trisilylalane Al(Si^*t*^Bu_2_Me)_3_ (1) and its corresponding radical anion 2[M] obtained by the reduction with an alkali metal. (c) Reported CO homologation by organoaluminium compounds. (d) This work: the isocyanide trisilylalane complexes 3–6 and the CO homologation product 7 mediated by radical anion 2[K].

The isocyanide trisilylalane complexes 3–5 were prepared by stirring the isocyanide with Al(Si^*t*^Bu_2_Me)_3_ (1) at room temperature in a solution of toluene (Scheme 1). Colorless crystals of compounds 3–5 suitable for single crystal X-ray diffraction (scXRD) analysis were grown from the saturated pentane solution at −30 °C. Although the poor quality of the molecular structure data for 3 precluded detailed structural analysis, molecular connectivity could still be established (Fig. S22, SI). Comprehensive structures of 4 and 5 are shown in Scheme 1. The C1–Al1 coordination bonds in 4 and 5 (2.093(1) and 2.086(2) Å) are longer than those of isocyanide triarylalane complexes and shorter than those of isocyanide trialkyl complexes.^7,8^ The N1–C1–Al1 skeleton in complex 5 is nearly linear with an angle of 177.3(2)°, while complex 4 shows a more bent structure (169.93(9)°).

**Scheme 1:**
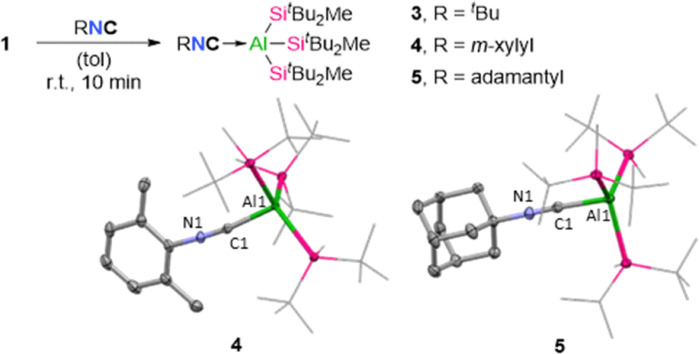
Synthesis of isocyanide alane complexes 3–5 and molecular structures of 4 and 5 (thermal ellipsoids are shown at a 50% probability level; H atoms are omitted for clarity). Selected bond lengths [Å] and angles [°]: for 4: C1–Al1 2.093(1), N1–C1–Al1 169.93(9); for 5: C1–Al1 2.086(2), N1–C1–Al1 177.3(2).

We also explored the reactivity of radical anion 2[K] with isocyanides. Interestingly, reactions of compound 2[K] with different isocyanides afforded the same complex 6 along with the cleavage of the R-NC single bond (Scheme 2). The loss of ^*t*^Bu as isobutylene, *m*-xylyl as *m*-xylene, and adamantyl as adamantane was corroborated by ^1^H NMR spectroscopy (Fig. S14–S16, SI). Crystals of 6 were obtained by storing its pentane solution at −30 °C for several days. Due to the high symmetry of the structure, a full refinement was not achieved. Nevertheless, the bonding arrangement could still be determined (Fig. S25, SI). The C–N bond cleavage and CN^−^ ion generation in the reactions of other organoaluminium compounds with ^*t*^BuNC were observed in previous studies.^24–26^ Similar reactivity has also been noted for other main group element compounds.^27–29^ In the present case, formation of 6 is proposed to proceed *via* single-electron transfer from radical anion 2[K] to RNC, followed by C–N bond cleavage of the resulting unstable [R-NC]˙^−^. The CN^−^ ion binds to the Al-center to give 6, while the R˙ radical undergoes H-abstraction from the solvent or β-elimination to afford the side product.

**Scheme 2:**
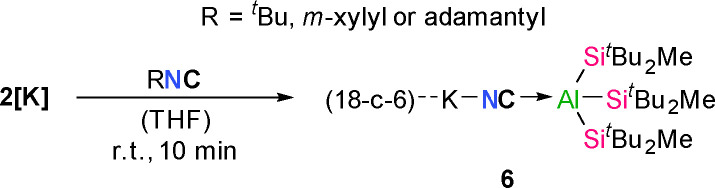
Synthesis of (18-c-6)KNC·Al(Si^*t*^Bu_2_Me)_3_ (6).

Next, the potential reactivity of compounds 1 and 2[K] with CO was investigated. While no isolable product was obtained from alane 1, exposure of a THF solution of 2[K] to excessive CO gas yielded compound 7 containing a C_3_ chain derived from two equivalents of M and three equivalents of CO with elimination of one silyl substituent (Scheme 3). CO homologation product 7 was purified *via* recrystallization from its THF solution. The solid-state structure was elucidated by scXRD and reveals a bicyclic [5,5] *ortho*-fused ring-shaped anion with a potassium counterion. The C2–C3, C1–C2 and C1–O2 distances (1.322(5), 1.380(7) and 1.291(7) Å) are considerably shorter than a typical C–C or C–O single bond, indicating a certain amount of double bond character and electron delocalization.^30^ The dative bonding nature of Al1–O2 and Al2–O1 is suggested by their elongated bond lengths (1.998(4) and 2.006(4) Å).

**Scheme 3:**
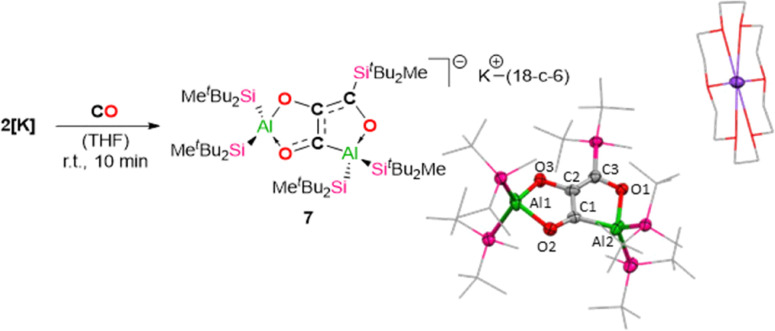
CO homologation mediated by radical anion 2[K] and the molecular structure of product 7 (thermal ellipsoids are shown at a 50% probability level; H atoms are omitted for clarity). Selected bond lengths [Å] and angles [°]: Al1–O3 1.718(4), Al1–O2 1.998(4), C2–O3 1.501(5), C1–O2 1.291(7), C2–C3 1.322(5), C1–C2 1.380(7), C3–O1 1.341(6), Al2–O1 2.006(4), Al2–C1 1.954(6).

Analysis of the crude reaction mixture of anion 2[K] with CO by ^1^H NMR spectroscopy revealed the formation of HSi^*t*^Bu_2_Me (Fig. S21, SI), consistent with loss of the silyl group. A comparable ring system was previously isolated as the side product from the reaction of alumylene with metal carbonyls.^19^ Based on the observed silyl elimination, we propose that CO homologation with 2[K] is initiated by Al-Si bond cleavage and subsequent interaction of the resulting aluminium center with CO, consistent with established pathways for aluminium-mediated CO homologation involving initial CO coordination and C–C bond formation.^17,19^

In summary, we isolated complexes 3–5 obtained from the coordination of isocyanides to trisilylalane 1. Reactions of Al(ii) radical anion 2[K] with different isocyanides afford the same compound 6 concomitant with the release of the alkyl or aryl groups, which likely proceeds *via* a single-electron pathway. Moreover, CO homologation mediated by 2[K] was demonstrated. Bicyclic compound 7 featuring a C_3_ chain was fully characterized. Taken together, the silyl-substituted Al(iii) species 1 and Al(ii) species 2[K] show promising potential in small molecule activation, and further studies on silyl-substituted aluminium compounds are ongoing in our research group.

## Conflicts of interest

There are no conflicts to declare.

## Supplementary Material

CC-062-D6CC02324J-s001

CC-062-D6CC02324J-s002

## Data Availability

The data supporting this article have been included in the supplementary information (SI). Supplementary information: experimental procedures and spectra. See DOI: https://doi.org/10.1039/d6cc02324j. CCDC 2543012 (4), 2543016 (5) and 2543019 for (7) contain the supplementary crystallographic data for this paper.^31*a*–*c*^
