# Supplementary material for: CO homologation and isocyanide activation by a trisilyl alane radical anion
Source: Chem Commun (Camb). 2026 Jun 8;62(52):13150–2. doi: 10.1039/d6cc02324j (PMC13273014; doi:10.1039/d6cc02324j)
Supplement: CC-062-D6CC02324J-s001 [file CC-062-D6CC02324J-s001.pdf]

Supplementary Information (SI) for ChemComm.
This journal is © The Royal Society of Chemistry 2026

Supporting Information

CO Homologation and Isocyanide Activation by a Trisilyl Alane Radical Anion

Yueer Zhu[1], Xufang Liu[1], Fiona J. Kiefer[1] and Shigeyoshi Inoue*[1]

[1] Department of Chemistry, TUM School of Natural Science, Technische Universität München, Lichtenbergstraße 4, 85375, Garching bei München, Germany

* E-mail: s.inoue@tum.de.

1 **General Methods and Instrumentation** **3**

2 **Synthesis and Characterization** **4**

2.1 $Al(Si^tBu_2Me)_3^-K^+$(18-c-6) (**2[K]**) 4

2.2 $^tBuNC{\cdot}Al(Si^tBu_2Me)_3$ (**3**) 5

2.3 $XylNC{\cdot}Al(Si^tBu_2Me)_3$ (**4**) 8

2.4 $AdNC{\cdot}Al(Si^tBu_2Me)_3$ (**5**) 11

2.5 (18-c-6)K-NC$\cdot Al(Si^tBu_2Me)_3$ (**6**) 14

2.6 CO homologation product **7** 20

3 **Single-Crystal X-ray Diffraction Analysis** **24**

3.1 General information 24

3.2 Molecular structure of **3** 26

3.3 Molecular structure of **4** 27

3.4 Molecular structure of **5** 29

3.5 Molecular structure of **6** 31

3.6 Molecular structure of **7** 32

4 **References** **34**

1 General Methods and Instrumentation

All experiments were performed in an argon 4.6 (≤ 99.996%; *Westfahlen AG*) atmosphere under the exclusion of H_2O and O_2 using standard Schlenk- or *LABstar* from *M. Braun Inertgas Systeme GmbH*. The glassware used for the reactions was heat-dried under vacuum before being sealed with Triboflon III grease. Solvent was purified via distillation in an inert gas atmosphere from sodium/ketyl radical, calcium hydride, or potassium mirror, and stored over molecular sieves (3 Å) under Schlenk conditions or in a Glovebox Workstation. Air- and moisture-sensitive compounds and reactions were measured in *J. Young* PTFE NMR tubes. Unless otherwise stated, all commercially available chemicals were purchased from *abcr GmbH* or *Sigma-Aldrich Chemie GmbH* and used without further purification. The starting materials HSi^tBu_2Me, $BrSi^tBu_2Me$, $NaSi^tBu_2Me$, $Al(Si^tBu_2Me)_3$ (**1**) were prepared according to the literature procedures.[1-4]

The NMR spectra were recorded at room temperature on a Bruker 500 MHz or a Bruker 400 MHz spectrometer. The δ-values are stated in parts per million [ppm]. Chemical shifts are referenced to (residual) solvent signal (C_6D_6: $\delta(^1H)$ = 7.16 ppm and $\delta(^{13}C)$ = 128.06 ppm).[5] The following abbreviations were used to assign the signals and those for the signal multiplicities: s-singlet, d-doublet, and sep-septet. Coupling constants are stated in Hertz [Hz].

Spectroscopy (LIFDI-MS) was measured directly from an inert atmosphere glovebox with a *Thermo Fisher Scientific Exactive Plus Orbitrap* equipped with an ion source from *Linden CMS*. HR-MS (ESI^+) analysis was performed on a *Thermo Fisher Scientific Exactive Plus Orbitrap* mass spectrometer equipped with a *Thermo Fischer* ESI source.

Quantitative elemental analyses (EA) were carried out using a *EURO EA (HEKA tech)* instrument equipped with a CHNS combustion analyzer at the Laboratory for Microanalysis at the TUM Catalysis Research Center.

2 Synthesis and Characterization

2.1 $Al(Si^tBu_2Me)_3^-K^+$(18-c-6) (**2[K]**)

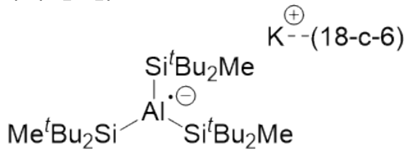

2[K]

KC_8 (104 mg, 769 µmol, 1.20 eq.) was added to a solution of $Al(Si^tBu_2Me)_3$ (**1**, 320 mg, 641 µmol, 1.00 eq.) in THF (10 mL). The reaction mixture was stirred at room temperature for ten hours, during which time it turned deep red. To monitor the reaction progress, ^1H NMR spectra of the mixture were collected during the reaction. Once the peaks corresponding to the starting material **1** had completely disappeared, the reaction was complete. The mixture was then filtered and the volatiles were removed under reduced pressure. 18-crown-6 (169 mg, 641 µmol, 1.00 eq.) and toluene (10 mL) were subsequently added to the resulting deep red solid. After the mixture was stirred, the solvent was removed under reduced pressure to give compound **2[K]** as a deep red powder. (442 mg, 551 µmol, 86%).

2.2 ^{t}BuNC·Al(Si^{t}Bu$_2$Me)$_3$ (**3**)

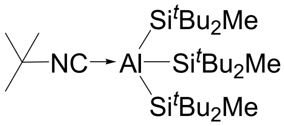

3

A solution of ^{t}BuNC (22.7 µL, 16.7 mg, 200 µmol, 1.00 eq.) in toluene (1 mL) was added to a solution of Al(Si^{t}Bu$_2$Me)$_3$ (**1**, 100 mg, 200 µmol, 1.00 eq.) in toluene (3 mL) at room temperature. The yellow solution decolorized immediately. After stirring for ten minutes, the solvent was removed under reduced pressure to give compound **3** as a white powder. (111 mg, 191 µmol, 95%). Suitable crystals for SC-XRD analysis were obtained by storing a saturated solution of **3** in pentane at −30 °C for several days.

^{1}H NMR (400 MHz, C_6D_6): δ [ppm] = 1.29 (s, 54H, Si^{t}Bu), 0.90 (s, 9H, NC^{t}Bu), 0.49 (s, 9H, SiMe).

^{13}C{^{1}H} NMR (126 MHz, C_6D_6): δ [ppm] = 58.37 (N*C*Me$_3$), 31.41 (SiC*Me$_3$*), 28.45 (NC*Me$_3$*), 22.69 (Si*C*Me$_3$), −0.39 (SiMe).

^{29}Si{^{1}H} NMR (99 MHz, C_6D_6): δ [ppm] = 13.68.

Melting point: 114 °C.

Elemental Analysis (%): Calcd: C 66.02, H 12.47, N 2.41; Found: C 65.14, H 12.05, N 2.54.

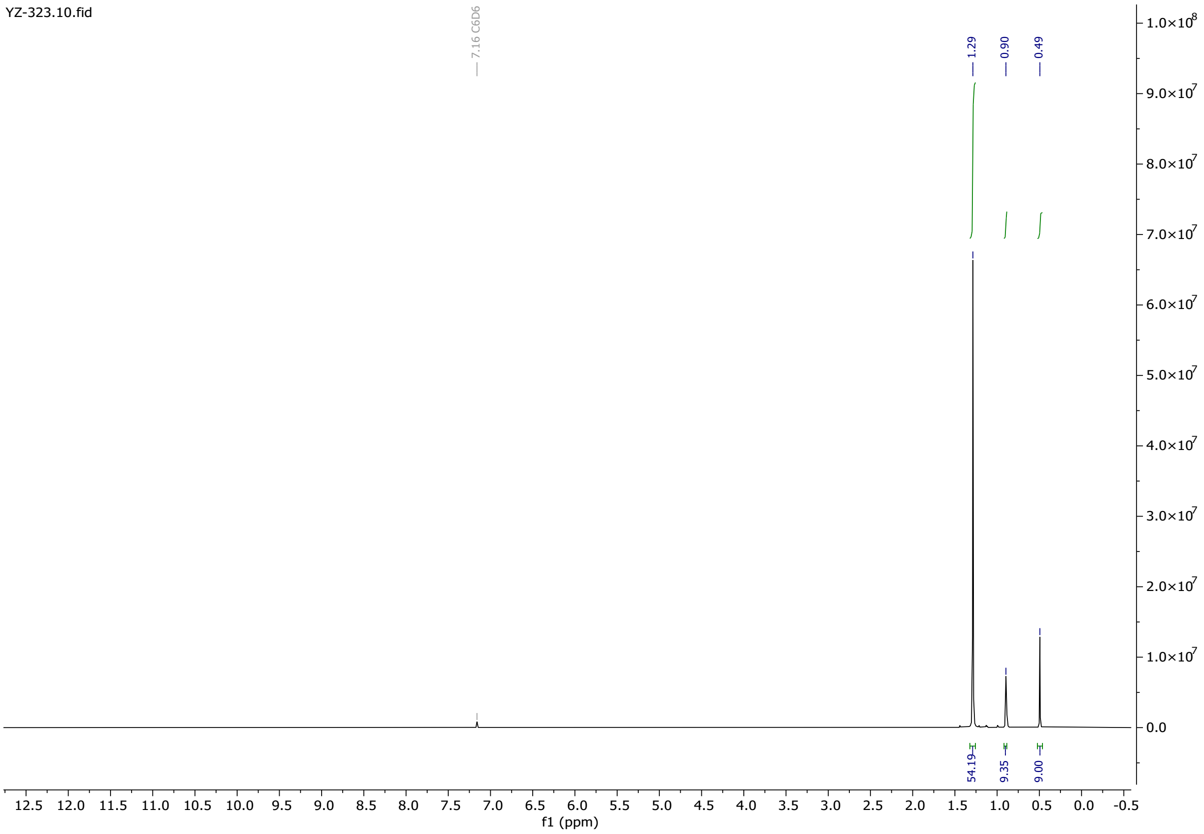

Figure S1: ^{1}H NMR spectrum of compound **3** in C_6D_6 at 298 K.

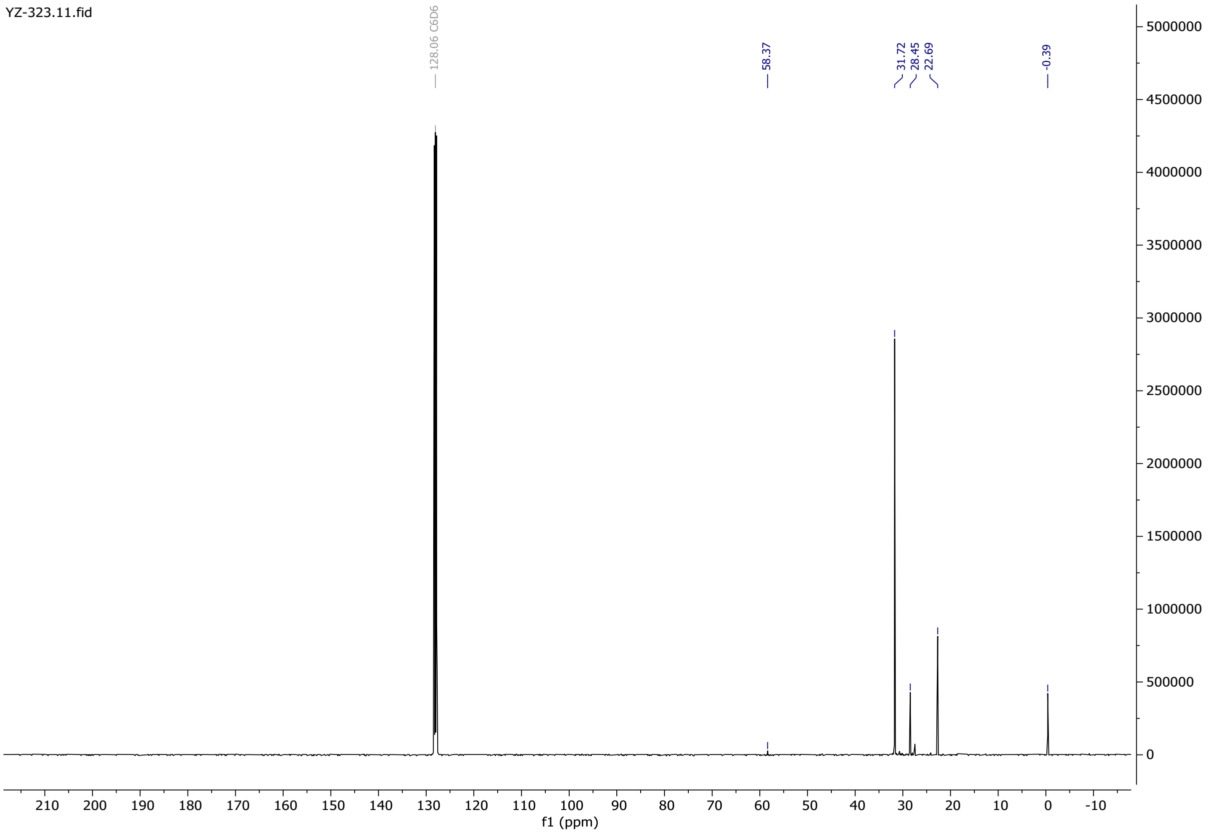

Figure S2: $^{13}C\{^{1}H\}$ NMR spectrum of compound **3** in C_6D_6 at 298 K.

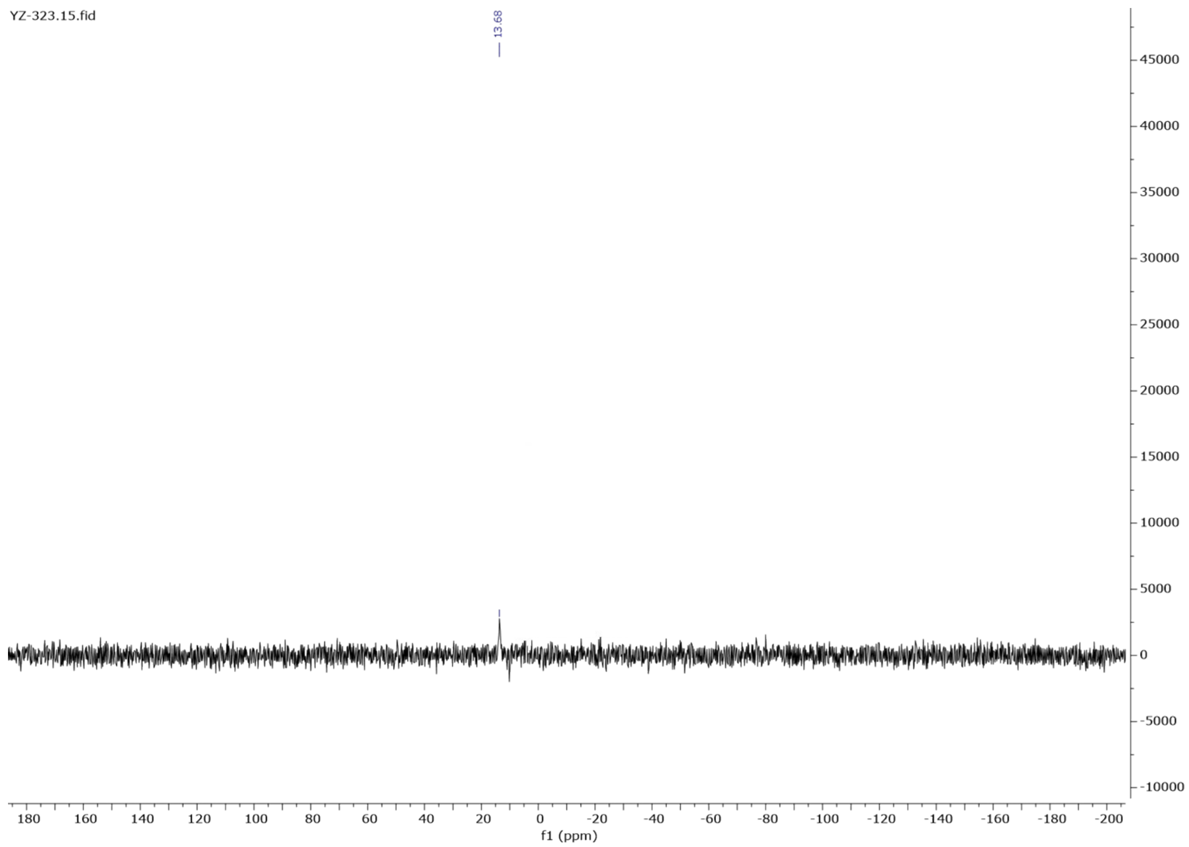

Figure S3: $^{29}Si\{^1H\}$ NMR spectrum of compound **3** in C_6D_6 at 298 K.

2.3 XylNC·Al(Si^{t}Bu$_2$Me)$_3$ (**4**)

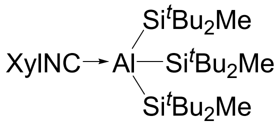

4

A solution of XylNC (26.3 mg, 200 µmol, 1.00 eq.) in toluene (1 mL) was added to a solution of Al(Si^{t}Bu$_2$Me)$_3$ (**1**, 100 mg, 200 µmol, 1.00 eq.) in toluene (3 mL) at room temperature. The yellow solution decolorized immediately. After stirring for ten minutes, the solvent was removed under reduced pressure to give compound **4** as a white powder. (122 mg, 194 µmol, 97%). Suitable crystals for SC-XRD analysis were obtained by storing a saturated solution of **4** in pentane at −30 °C for several days.

^{1}H NMR (400 MHz, C_6D_6): δ [ppm] = 6.68-6.64 (m, 1H, *p*-H), 6.48 (d, *J* = 7.6 Hz, 2H, *m*-H), 2.25 (s, 6H, C_{aryl}Me), 1.33 (s, 54H, ^{t}Bu), 0.09 (s, 6H, SiMe).

^{13}C{^{1}H} NMR (126 MHz, C_6D_6): δ [ppm] = 136.20 (*o*-C), 130.59 (*p*-C), 128.92 (*m*-C), 32.15 (SiC*Me$_3$*), 23.25 (Si*C*Me$_3$), 19.53 (C_{aryl}*Me*), 0.09 (SiMe).

^{29}Si{^{1}H} NMR (99 MHz, C_6D_6): δ [ppm] = 14.42.

Melting point: 121 °C.

Elemental Analysis (%): Calcd: C 68.61, H 11.52, N 2.22; Found: C 69.08, H 11.07, N 2.42.

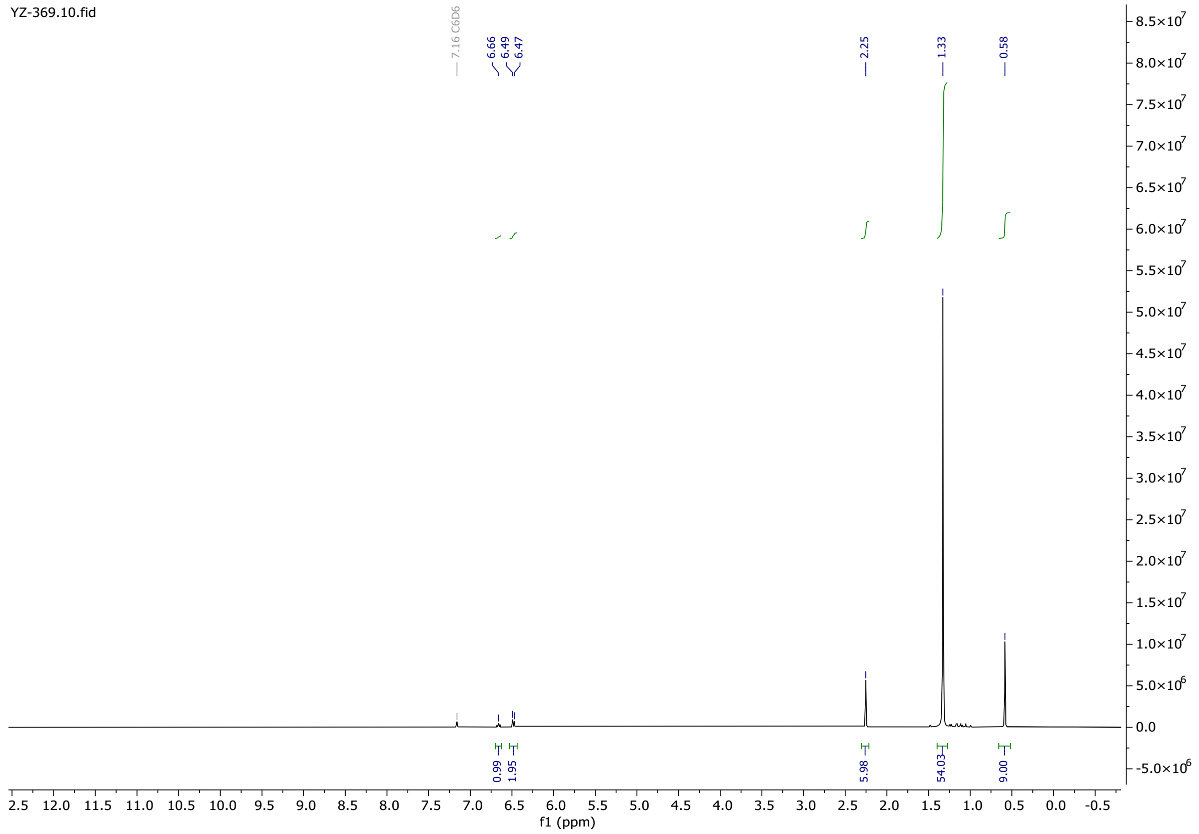

Figure S4: ^{1}H NMR spectrum of compound **4** in C_6D_6 at 298 K.

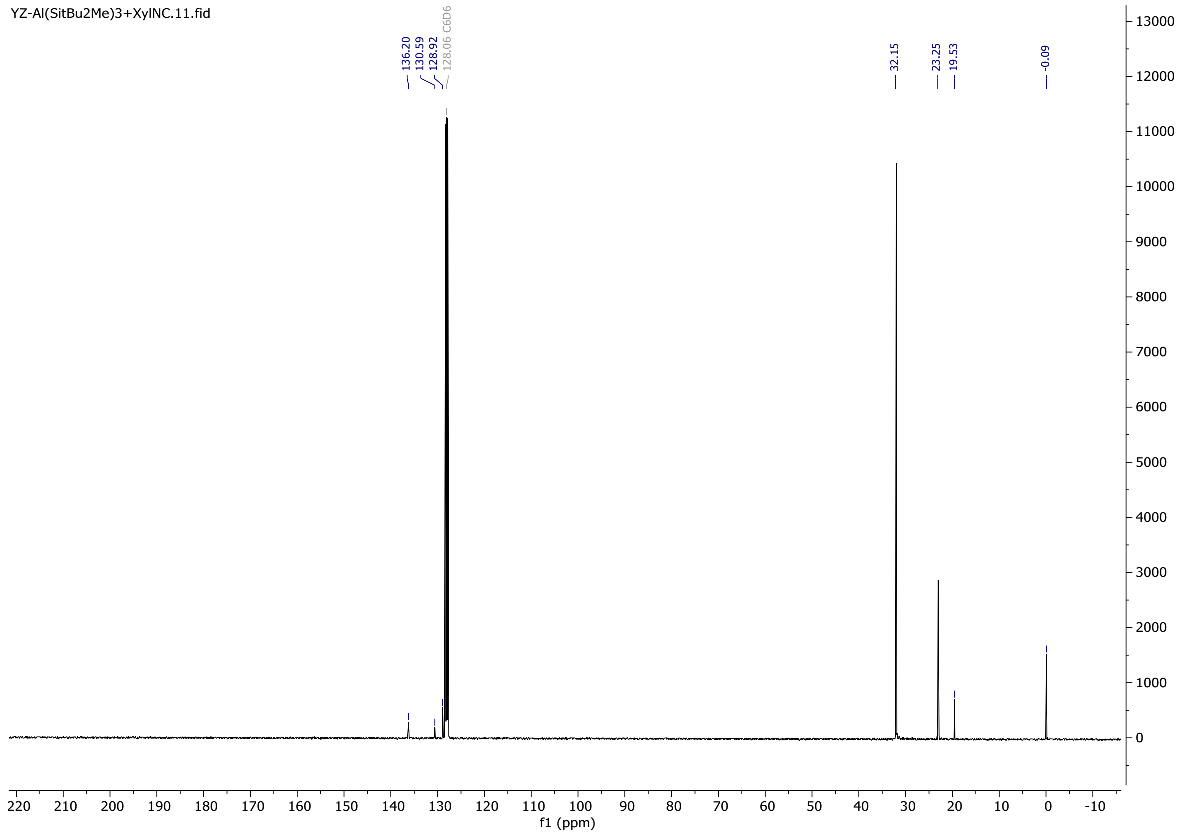

Figure S5: $^{13}C\{^{1}H\}$ NMR spectrum of compound **4** in C_6D_6 at 298 K.

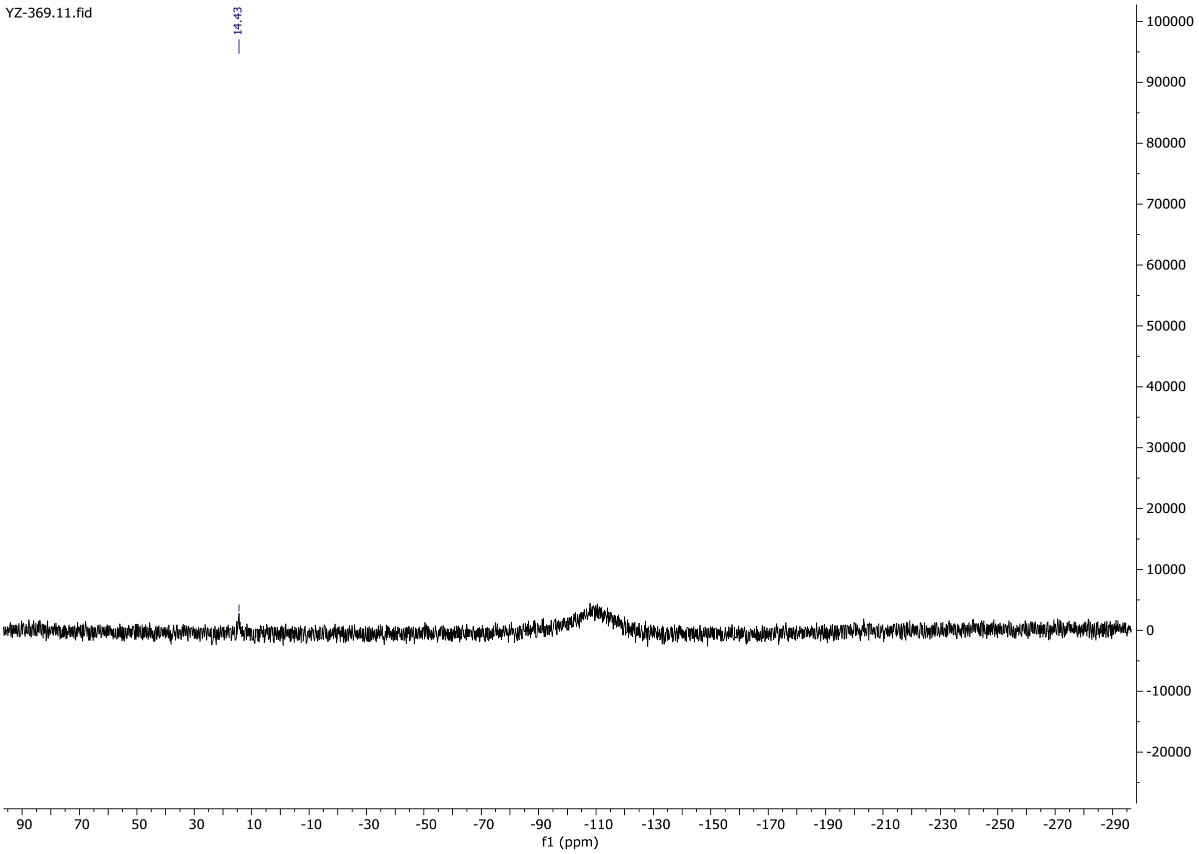

Figure S6: $^{29}Si\{^1H\}$ NMR spectrum of compound **4** in C_6D_6 at 298 K.

2.4 AdNC·Al(Si^{t}Bu$_2$Me)$_3$ (**5**)

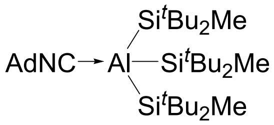

5

A solution of AdNC (32.3 mg, 200 µmol, 1.00 eq.) in toluene (1 mL) was added to a solution of Al(Si^{t}Bu$_2$Me)$_3$ (**1**, 100 mg, 200 µmol, 1.00 eq.) in toluene (3 mL) at room temperature. The yellow solution decolorized immediately. After stirring for ten minutes, the solvent was removed under reduced pressure to give compound **5** as a white powder. (128 mg, 194 µmol, 97%). Suitable crystals for SC-XRD analysis were obtained by storing a saturated solution of **5** in pentane at −30 °C for several days.

^{1}H NMR (400 MHz, CDCl$_3$): δ [ppm] = 2.16 (s, 3H, C_c), 2.07 (s, 6H, C_b), 1.74-1.65 (m, 6H, C_d), 1.05 (s, 54H, Si^{t}Bu), 0.19 (s, 6H, Me).

^{13}C{^{1}H} NMR (126 MHz, CDCl$_3$): δ [ppm] = 58.56 (C_a), 41.69 (C_b), 35.31(C_d), 31.46 (SiC*Me$_3$*), 28.58 (C_c), 22.49 (Si*C*Me$_3$), 0.88 (Me).

^{29}Si{^{1}H} NMR (99 MHz, CDCl$_3$): δ [ppm] = 13.34.

Melting point: 142 °C.

Elemental Analysis (%): Calcd: C 69.12, H 11.91, N 2.12; Found: C 66.27, H 13.34, N 1.92.

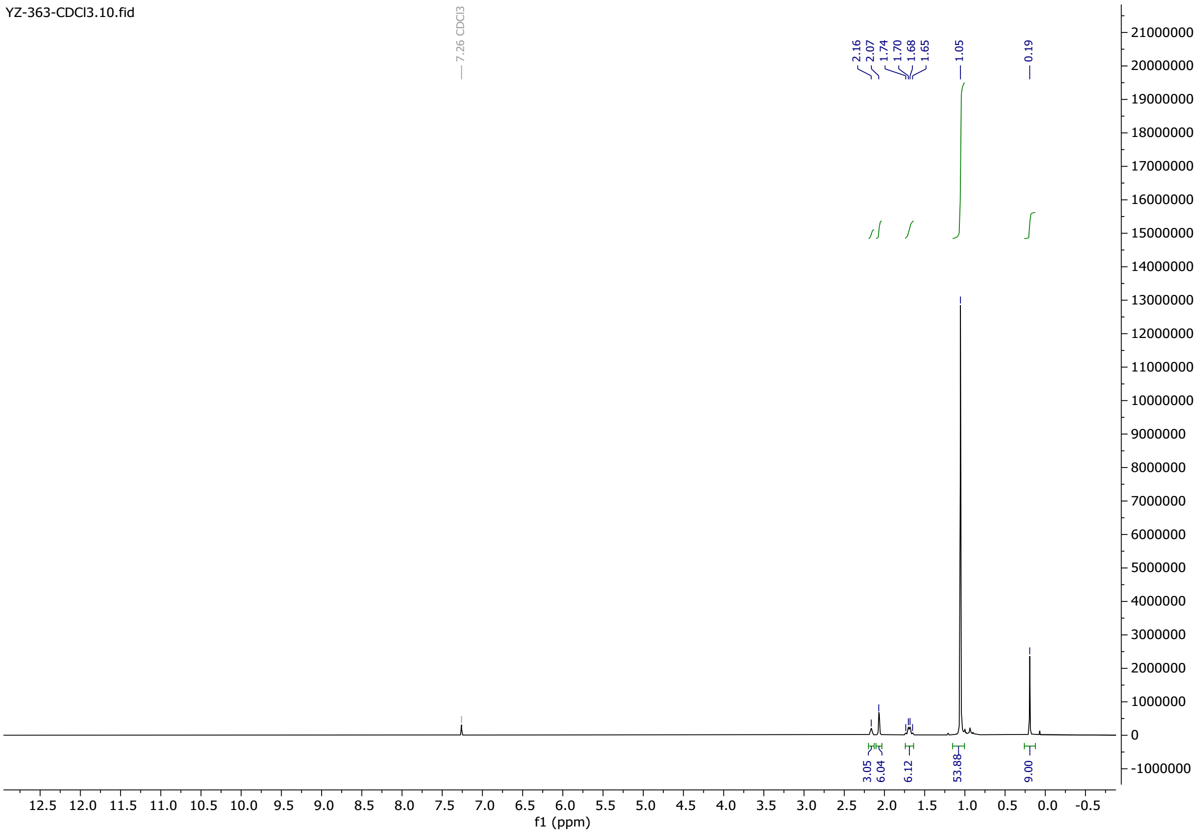

Figure S7: ^{1}H NMR spectrum of compound **5** in $CDCl_3$ at 298 K.

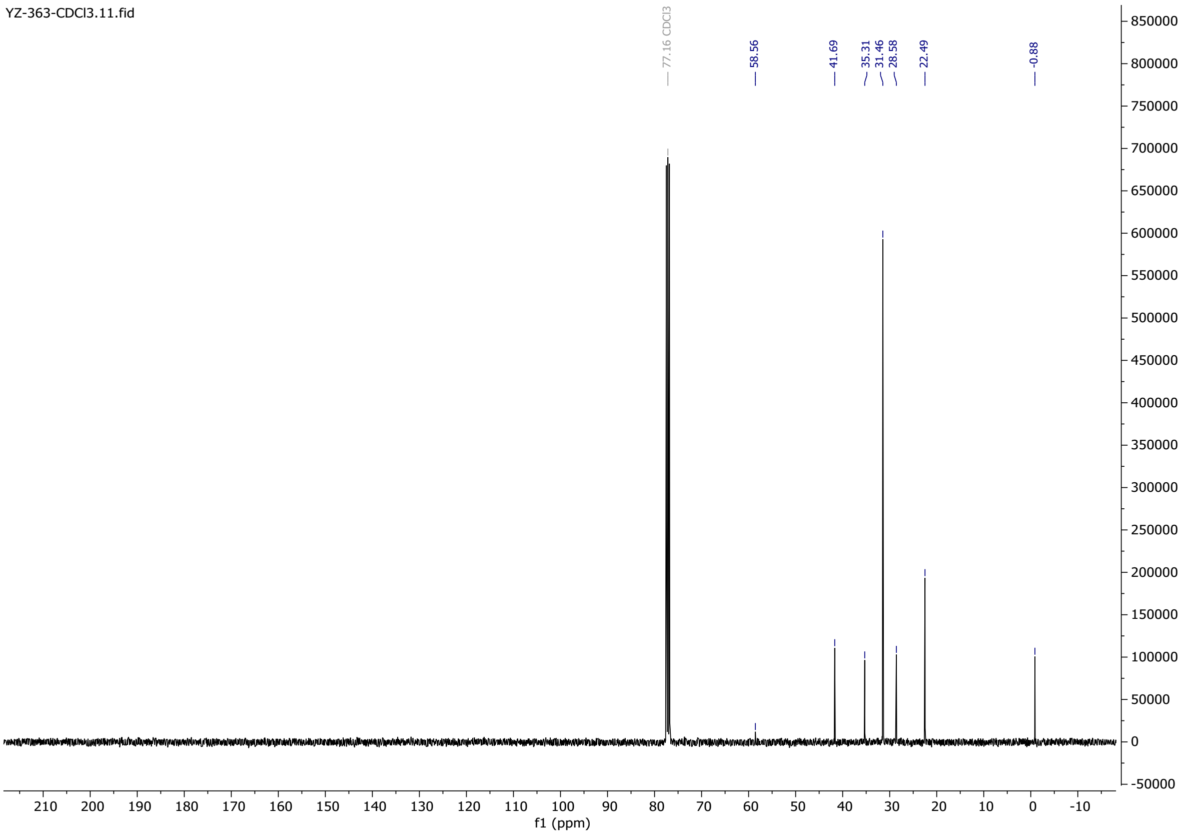

Figure S8: $^{13}C\{^{1}H\}$ NMR spectrum of compound **5** in $CDCl_3$ at 298 K.

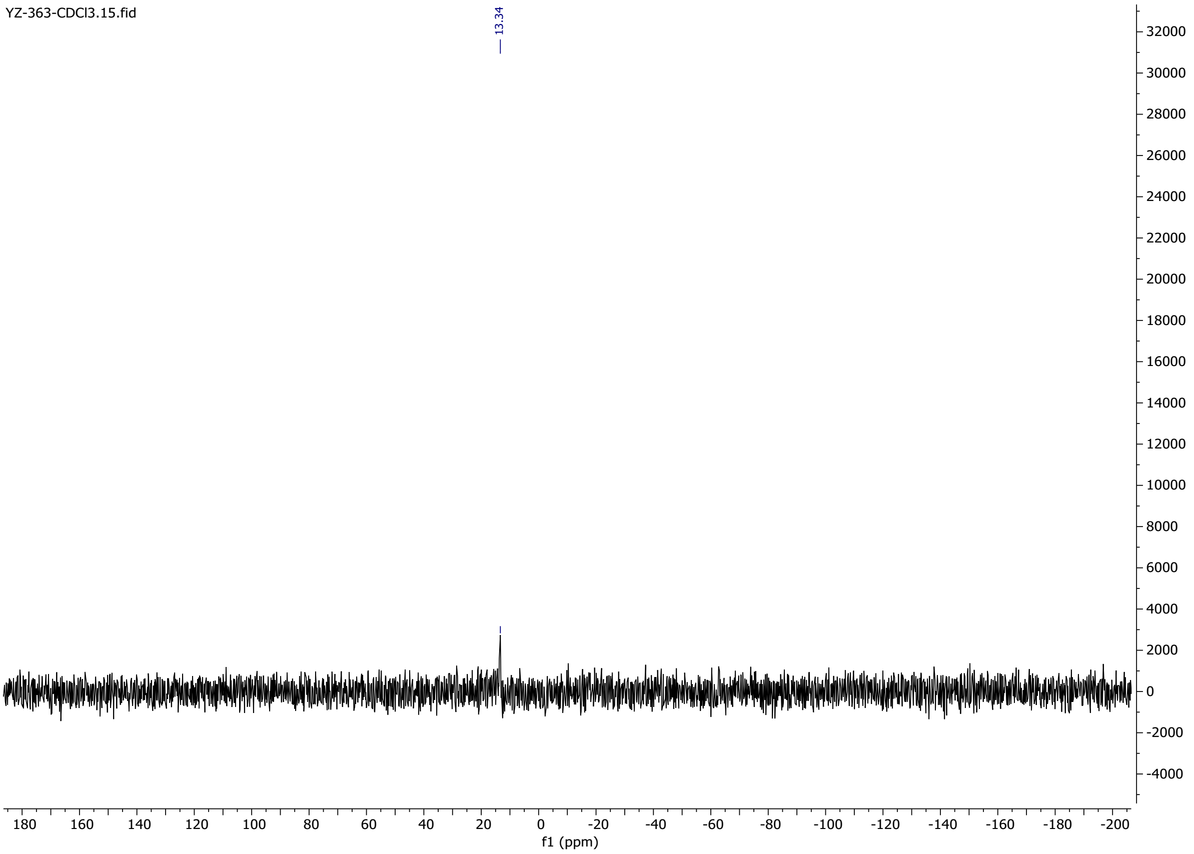

Figure S9: $^{29}Si\{^{1}H\}$ NMR spectrum of compound **5** in $CDCl_3$ at 298 K.

2.5 (18-c-6)K-NC·Al(Si^{t}Bu$_2$Me)$_3$ (**6**)

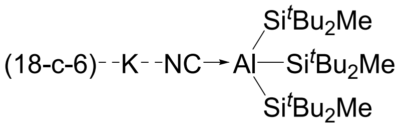

6

A solution of RNC (R = ^{t}Bu, 14.1 µL, 10.4 mg, 125 µmol, 1.00 eq.; R = Xyl, 16.4 mg, 125 µmol, 1.00 eq.; R = Ad, 20.1 mg, 125 µmol, 1.00 eq) in toluene (1 mL) was added to a solution of Al(Si^{t}Bu$_2$Me)$_3^-$K$^+$(18-c-6) (**2[K]**, 100 mg, 125 µmol, 1.00 eq.) in THF (3 mL) at room temperature. The deep red solution decolorized immediately. After stirring for ten minutes, the solvent was removed under reduced pressure to give compound **6** as a white powder. (R = ^{t}Bu, 96.1 mg, 116 µmol, 93%; R = Xyl, 95.2 mg, 115 µmol, 92%; R = Ad, 96.9 mg, 117 µmol, 94%). Suitable crystals for SC-XRD analysis were obtained by storing a saturated solution of **6** in pentane at −30 °C for several days.

^{1}H NMR (400 MHz, C$_6$D$_6$): δ [ppm] = 3.04 (s, 24H, 18-c-6), 1.55 (s, 54H, Si^{t}Bu), 0.74 (s, 6H, Me).

^{13}C{^{1}H} NMR (126 MHz, C$_6$D$_6$): δ [ppm] = 69.95 (18-c-6), 32.20 (SiC*Me$_3$*), 22.89 (Si*C*Me$_3$), −0.20 (Me).

^{29}Si{^{1}H} NMR (99 MHz, C$_6$D$_6$): δ [ppm] = 10.20.

LIFDI-MS (positive ion mode): m/z found: 303.1176 [$C_{12}H_{24}KO_6$]$^+$, Anion wasn't detected because of positive ion mode.

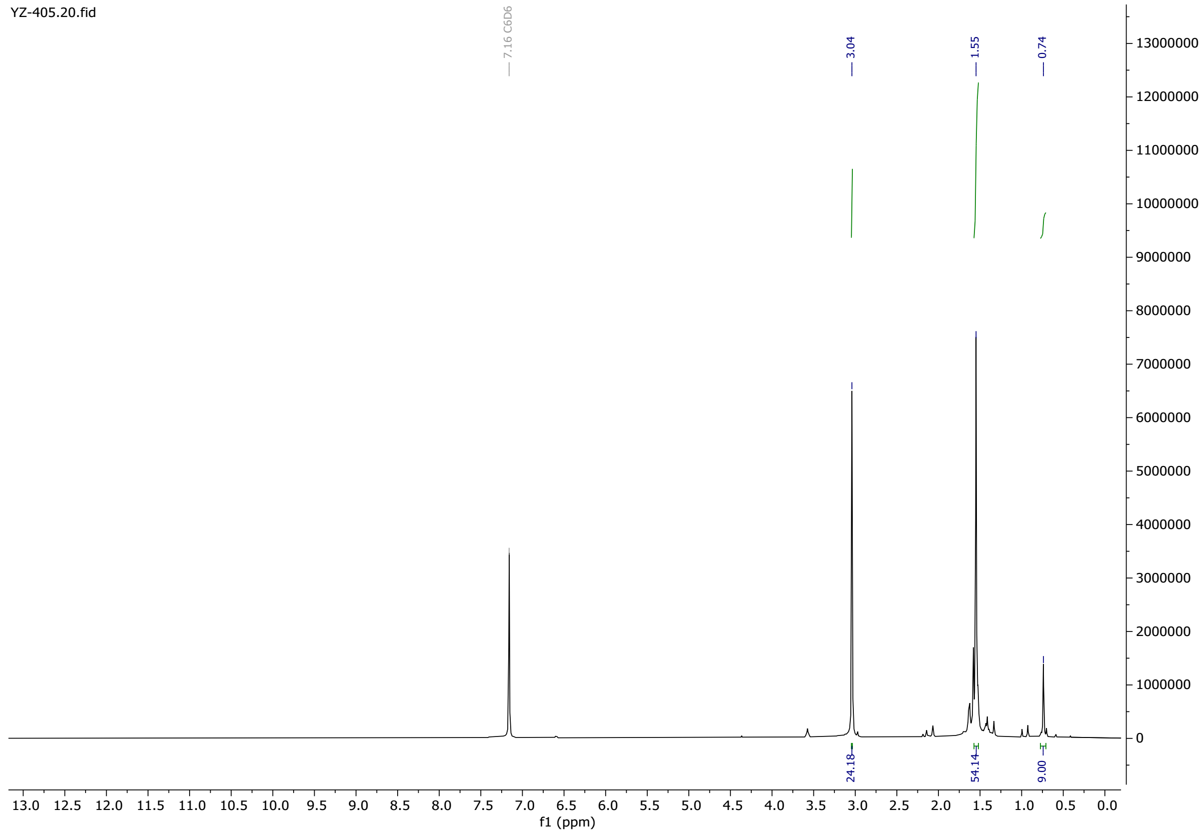

Figure S10: ^{1}H NMR spectrum of compound **6** in C_6D_6 at 298 K.

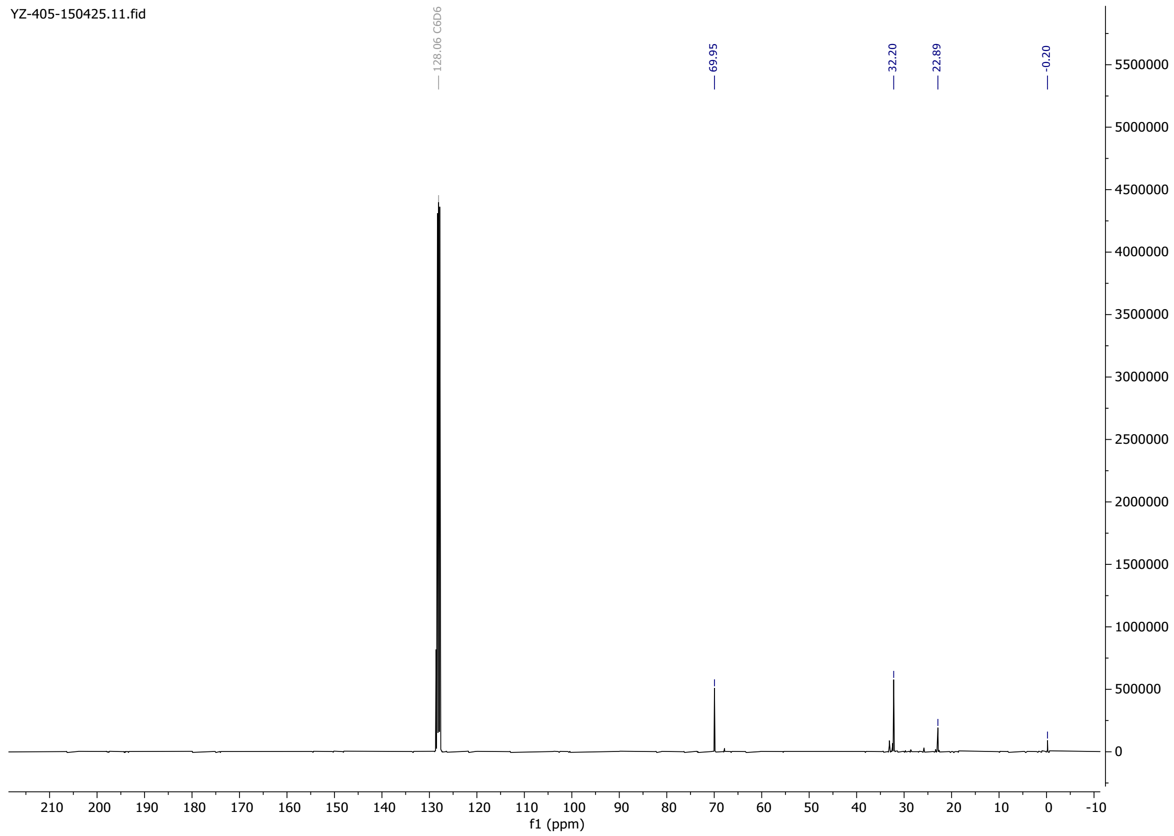

Figure S11: $^{13}C\{^1H\}$ NMR spectrum of compound **6** in C_6D_6 at 298 K.

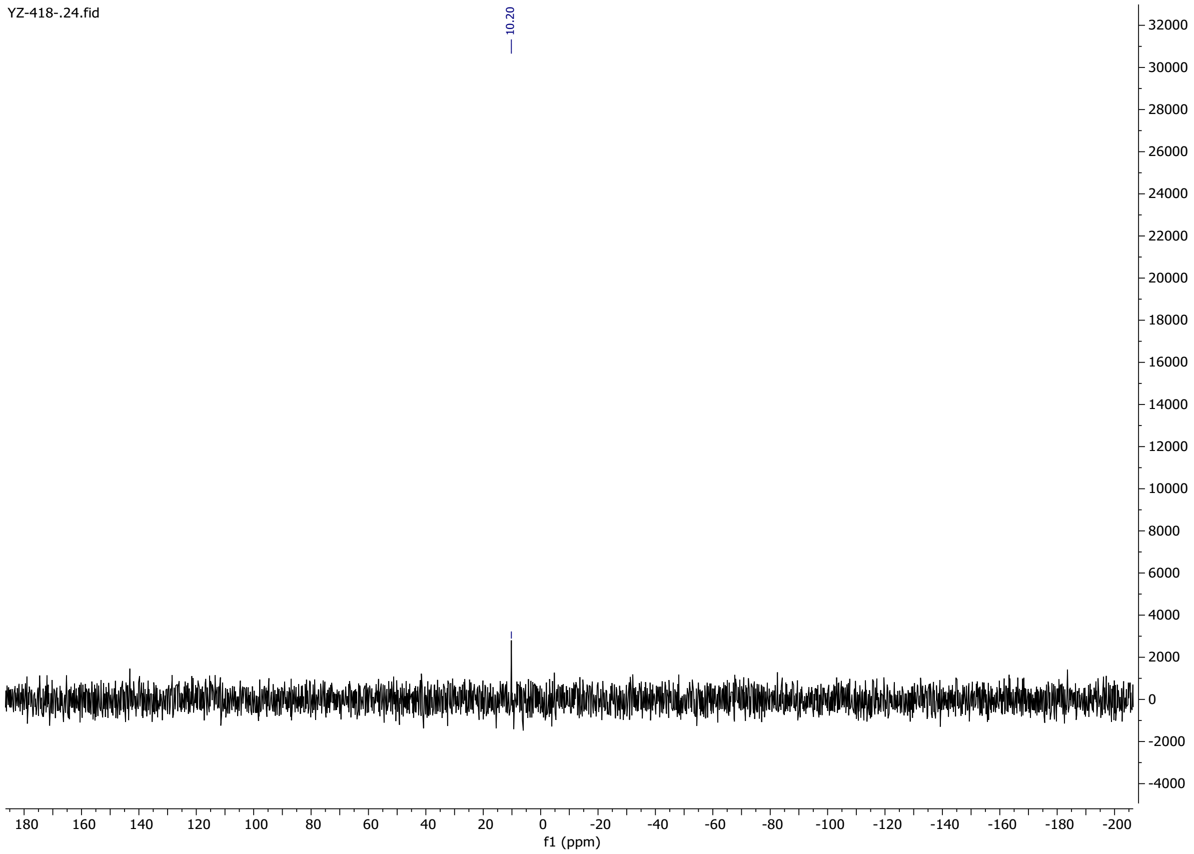

Figure S12: $^{29}Si\{^1H\}$ NMR spectrum of compound **6** in C_6D_6 at 298 K.

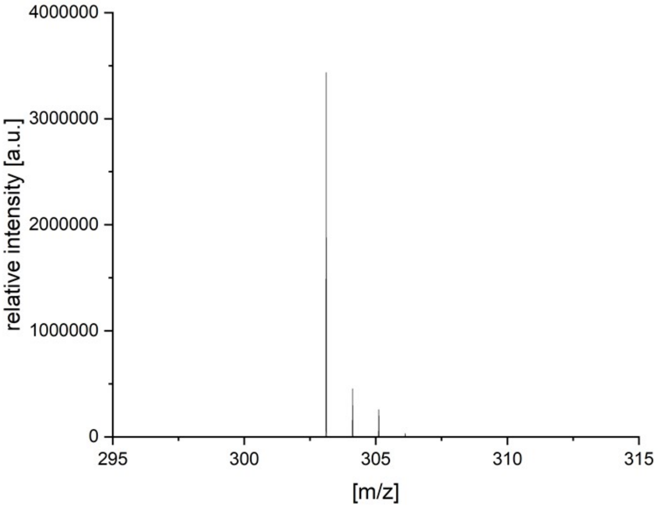

Figure S13: LIFDI-MS spectrometry (detail view with isotopic pattern) of **6**. Measured m/z = 303.1176 $[C_{12}H_{24}KO_6]^+$.

^{1}H NMR spectra of the reaction mixture of **2[K]** and *t*BuNC

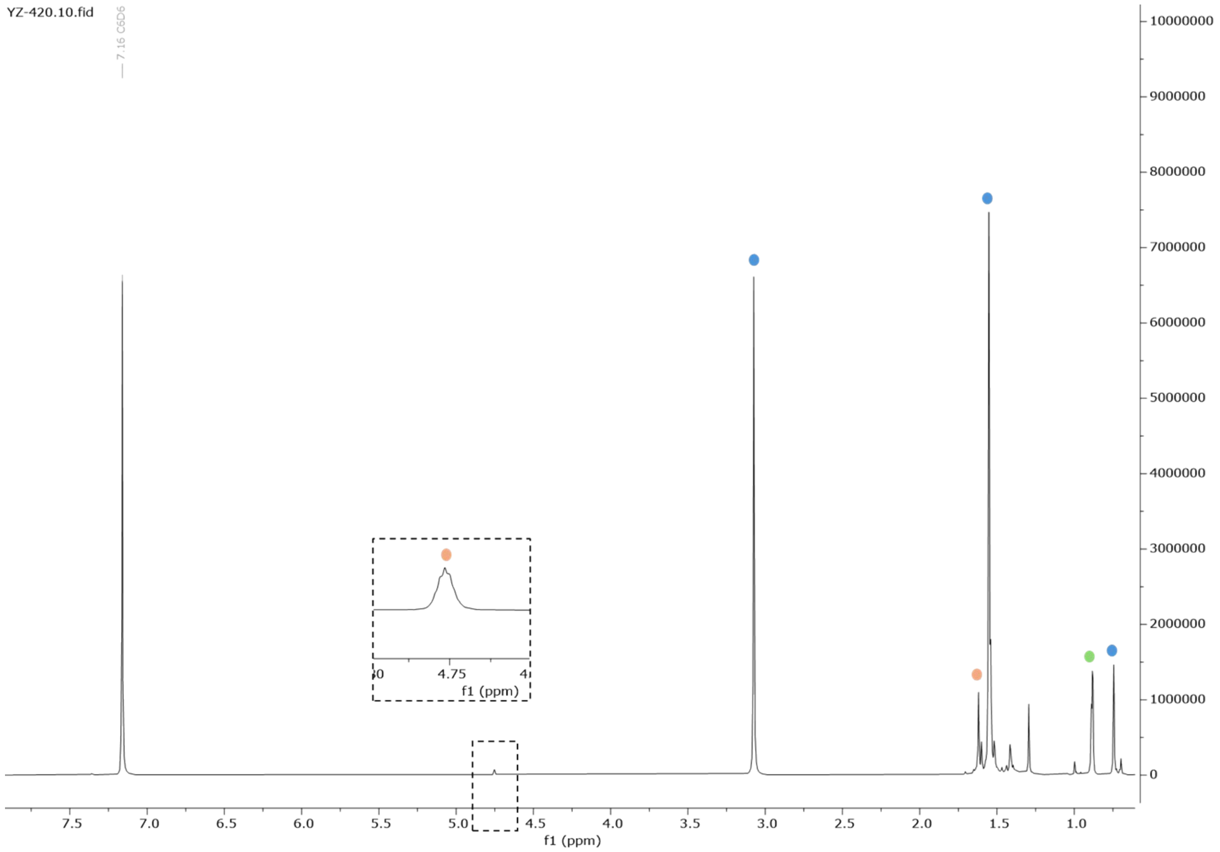

Figure S14: ^{1}H NMR spectrum of the reaction mixture of **2[K]** and *t*BuNC (blue: **6**; orange: isobutylene; green: *t*BuNC).

<u>^{1}H NMR spectra of the reaction mixture of **2[K]** and XylNC</u>

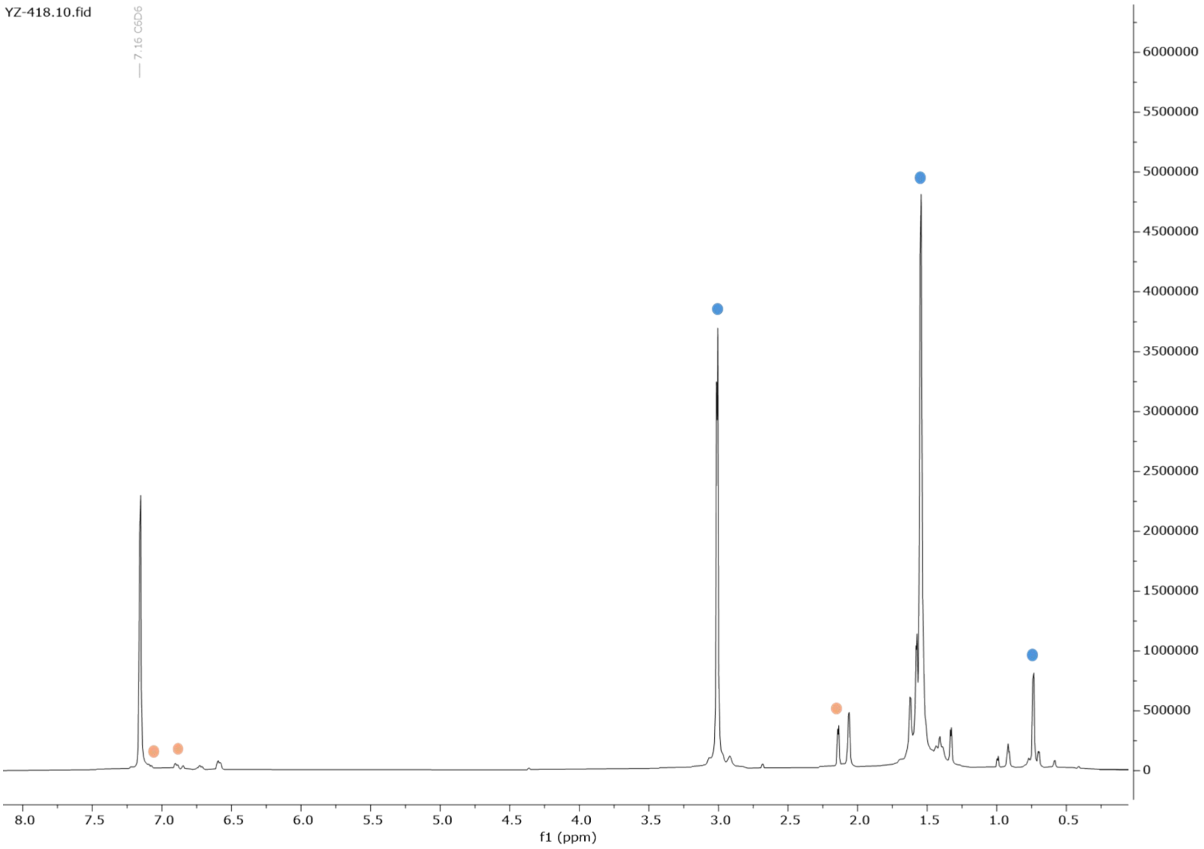

Figure S15: ^{1}H NMR spectrum of the reaction mixture of **2[K]** and XylNC (blue: **6**; orange: *m*-xylene).

^{1}H NMR spectra of the reaction mixture of **2[K]** and AdNC

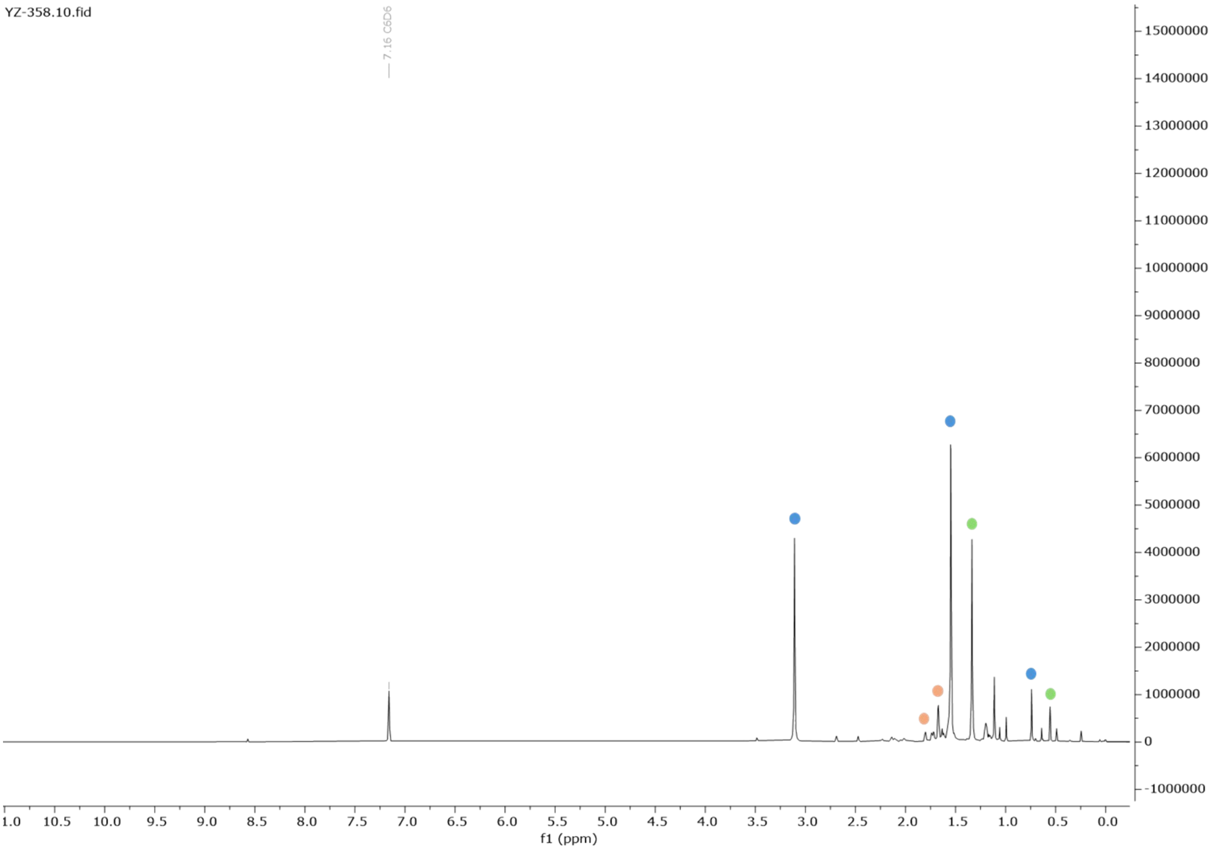

Figure S16: ^{1}H NMR spectrum of the reaction mixture of **2[K]** and AdNC (blue: **6**; orange: adamantane; green: $Al(Si^{t}Bu_2Me)_3$).

2.6 CO homologation product **7**

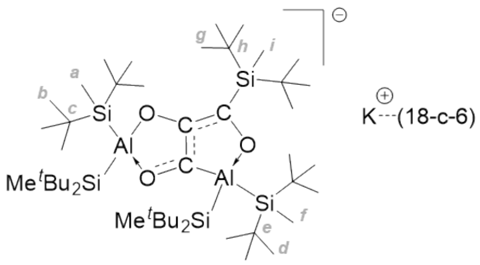

7

A degassed solution of Al(Si^{t}Bu$_2$Me)$_3^-$K$^+$(18-c-6) (**2[K]**, 25.0 mg, 31.2 µmol, 1.00 eq.) in THF in a *J. Young* NMR tube was pressed with CO at room temperature. The deep red solution turned orange. The reaction mixture was concentrated and stored at −30 °C for several days to give compound **7** as an orange crystal (7.51 mg, 6.11 µmol, 39%). The crystals are suitable for SC-XRD analysis.

^{1}H NMR (400 MHz, THF-d_8): δ [ppm] = 3.62 (s, 24H, 18-c-6), 1.12 (s, 18H, H^g), 1.00-0.98 (m, 72H, H^b and H^d), 0.27 (s, 3H, H^i), −0.02 (s, 12H, H^b and H^f).

^{13}C{^{1}H} NMR (126 MHz, THF-d_8): δ [ppm] = 192.48 (s, C^j), 175.56 (s, C^k), 71.39 (s, 18-c-6), 31.87 (d, J = 11.3 Hz, C^b), 31.58 (d, J = 14.5 Hz, C^d), 30.30 (s, C^g), 22.13 (d, J = 11.3 Hz, C^e), 21.69 (s, C^c), 20.79 (s, C^h), −3.51 (s, C^a), −4.03 (s, C^f), −6.87 (s, C^i).

^{29}Si{^{1}H} NMR (99 MHz, THF-d_8): δ [ppm] = 5.06 (s, C^fSi), 4.47 (s, C^jSi), −4.42 (s, C^aSi). (Signals were assigned using the ^{29}Si-HMBC spectrum.)

LIFDI-MS (positive ion mode): m/z found: 303.1203 [$C_{12}H_{24}KO_6$]$^+$, Anion wasn't detected because of positive ion mode.

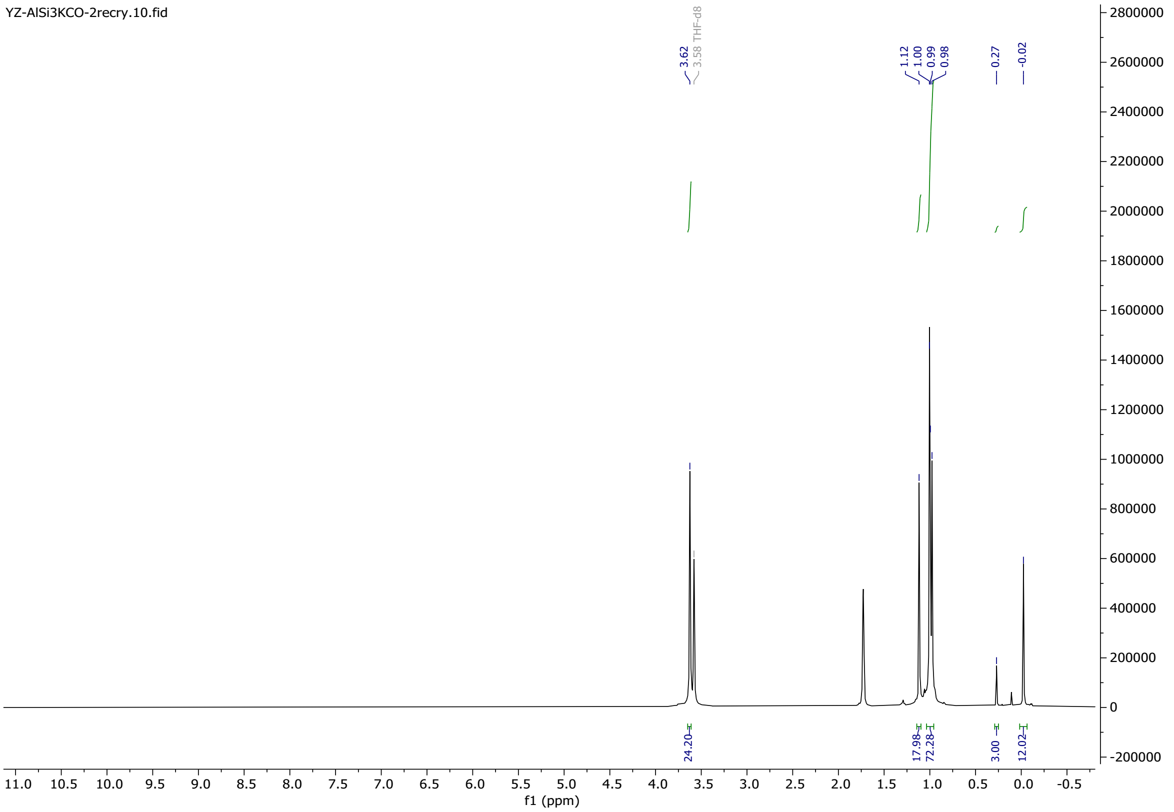

Figure S17: ^{1}H NMR spectrum of compound **7** in THF-d_8 at 298 K.

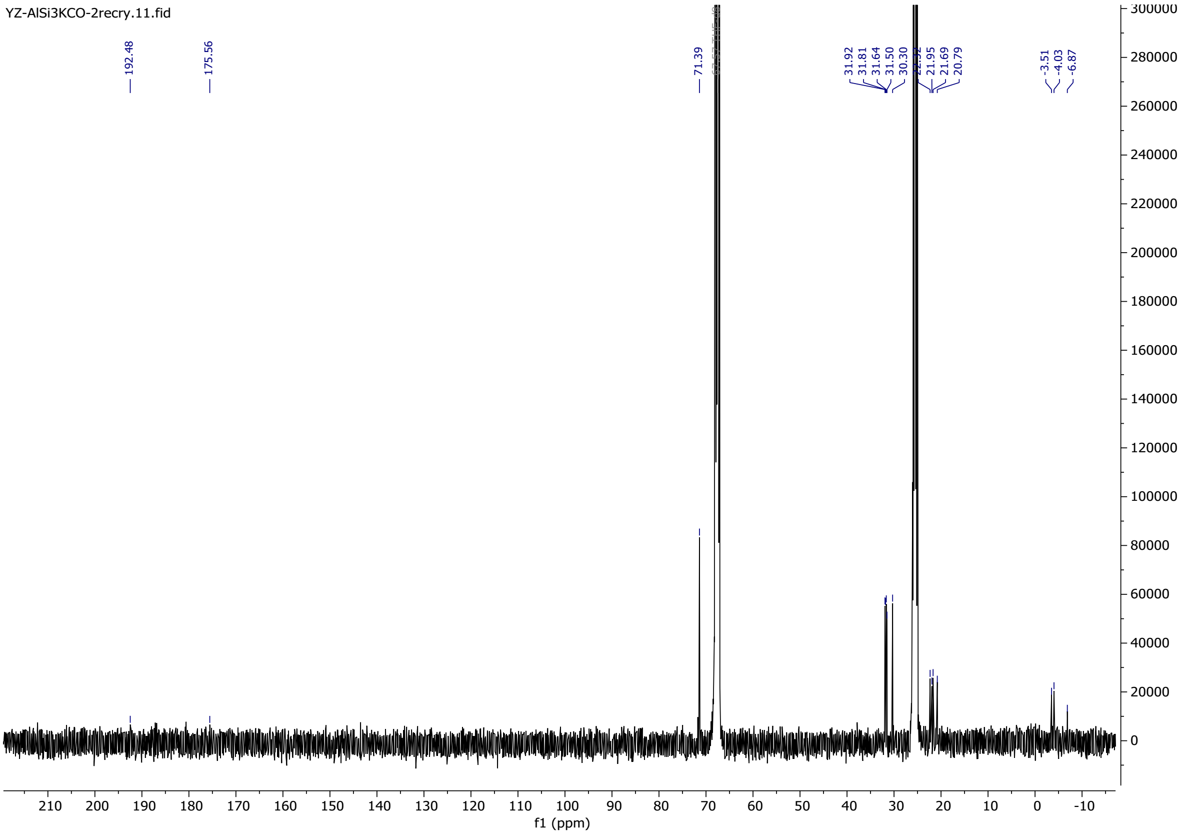

Figure S18: $^{13}C\{^{1}H\}$ NMR spectrum of compound **7** in THF-d_8 at 298 K.

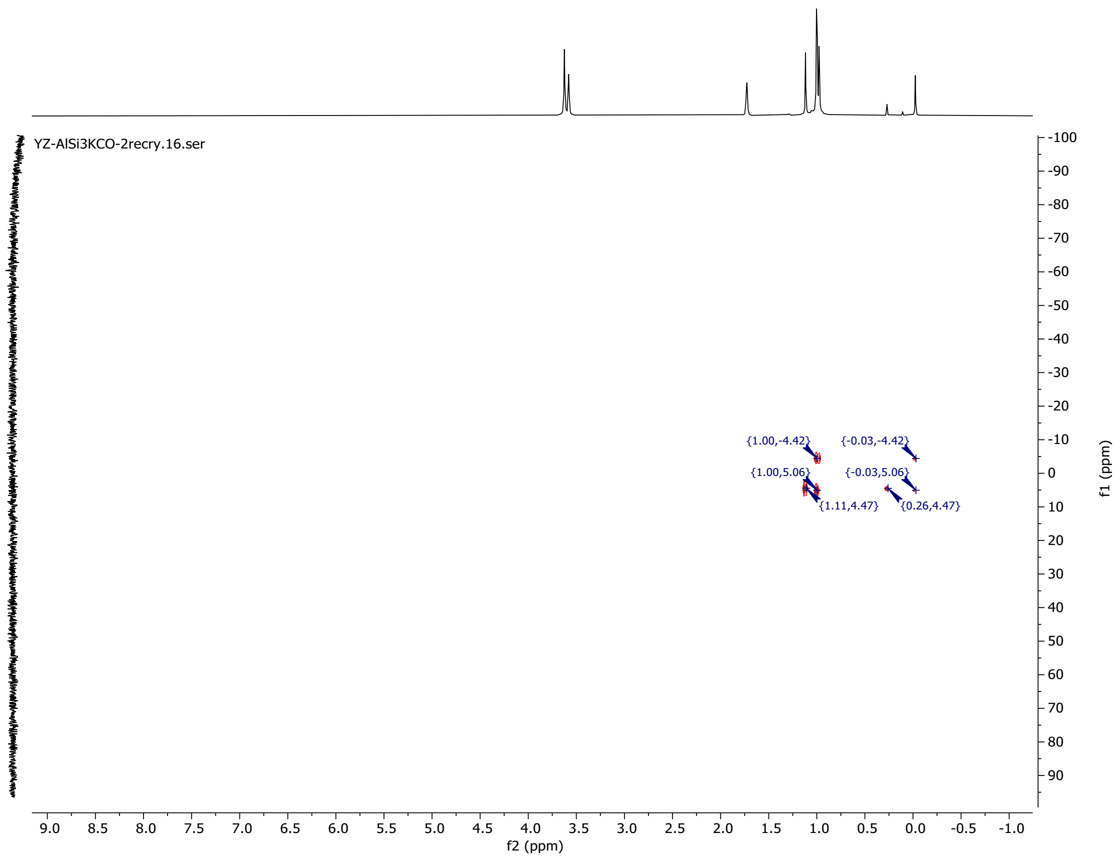

Figure S19: $^{29}Si\{^{1}H\}$ HMBC spectrum of compound **7** in THF-d_8 at 298 K.

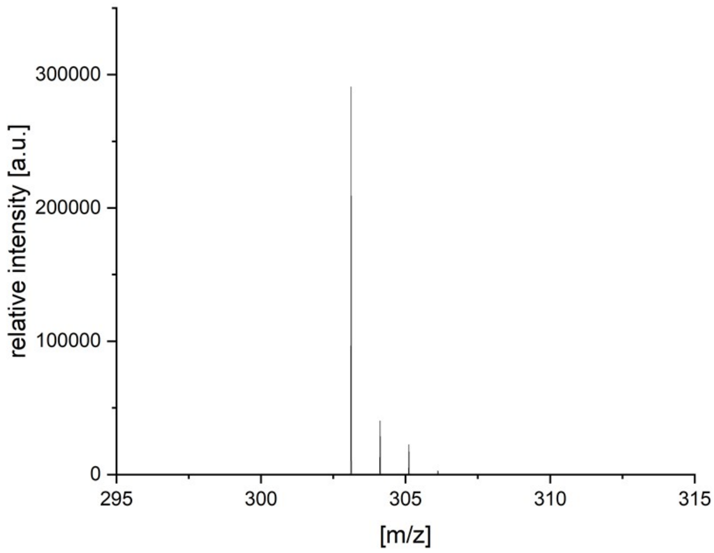

Figure S20: LIFDI-MS spectrometry (detail view with isotopic pattern) of **7**. Measured m/z = 303.1203 $[C_{12}H_{24}KO_6]^+$.

^1H NMR spectra of the crude reaction mixture of **2[K]** and CO

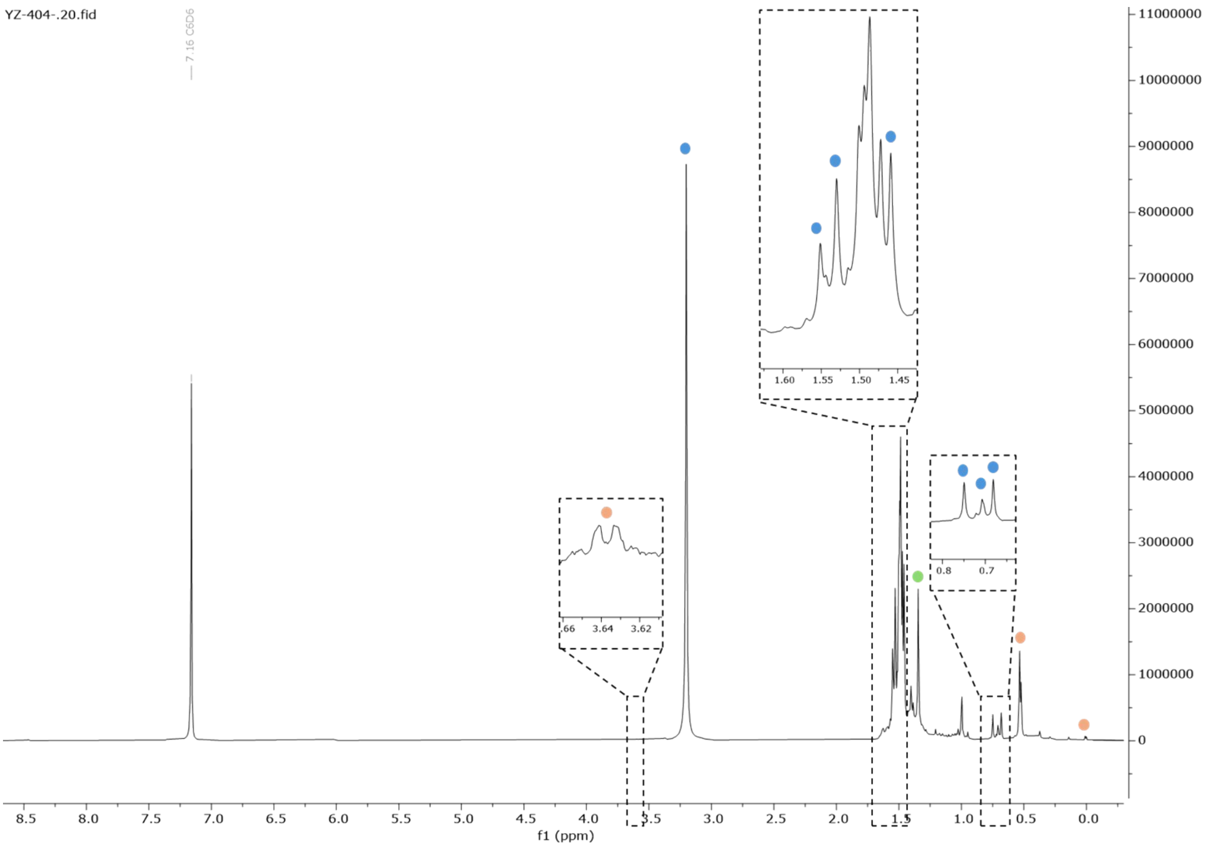

Figure S21: ^1H NMR spectrum of the crude reaction mixture of **2[K]** and CO (blue: compound **7**; orange: HSi^tBu_2Me, green: $Al(Si^tBu_2Me)_3$ (**1**)).

3 Single-Crystal X-ray Diffraction Analysis

3.1 General information

The X-ray intensity data were collected on an X-ray single crystal diffractometer equipped with a CMOS detector (Bruker Photon-100), a rotating anode (Bruker TXS) with MoKα radiation (λ = 0.71073 Å) and a Helios mirror optic by using the APEX4 software package or an X-ray single crystal diffractometer equipped with a CMOS detector (Bruker Photon-100), an IMS microsource with MoKα radiation (λ = 0.71073 Å) and a Helios mirror optic by using the APEX4 software package.[6] The measurement was performed on single crystals coated with perfluorinated ether. The crystal was fixed on the top of a microsampler, transferred to the diffractometer and measured under a stream of cold nitrogen. A matrix scan was used to determine the initial lattice parameters. Reflections were merged and corrected for Lorenz and polarization effects, scan speed, and background using SAINT. Absorption corrections, including odd and even ordered spherical harmonics, were performed using SADABS.[7] Space group assignments were based on systematic absences, E statistics, and successful refinement of the structures. Structures were solved by direct methods with the aid of successive difference Fourier maps and were refined against all data using the APEX4[8] in conjunction with SHELXL-2018/3.[9, 10] and SHELXLE.[11] Methyl hydrogen atoms were refined as part of rigid rotating groups, with a C–H distance of 0.98 Å and Uiso(H) = 1.5·Ueq(C). Other H atoms were placed in calculated positions and refined using a riding model, with methylene and aromatic C–H distances of 0.99 and 0.95 Å, respectively, and Uiso(H) = 1.2·Ueq(C). If not mentioned otherwise, non-hydrogen atoms were refined with anisotropic displacement parameters. Full-matrix least-squares refinements were carried out by minimizing $\Delta w(Fo^2\text{-}Fc^2)^2$ with SHELXL-97[12] weighting scheme. Neutral atom scattering factors for all atoms and anomalous dispersion corrections for the non-hydrogen atoms were taken from International Tables for Crystallography.[13] Refinement afforded using the function SQUEEZE/Platon[14], a volume of 241 $Å^3$ in 1 void per unit cell for one benzene molecule per asymmetric unit was found. Images of the crystal structures were generated by PLATON and MERCURY.[15, 16] The CCDC numbers CCDC 2543012, 2543016 and 2543019 contain the supplementary

crystallographic data for structure **4**, **5** and **7**. These data can be obtained free of charge from the Cambridge Crystallographic Data Centre via https://www.ccdc.cam.ac.uk/structures/. The CIF files were generated using FinalCif.[17]

3.2 Molecular structure of **3**

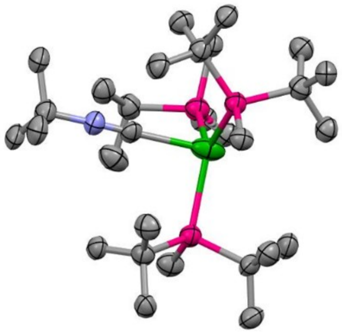

Figure S22: Connectivity of **3**.

3.3 Molecular structure of **4**

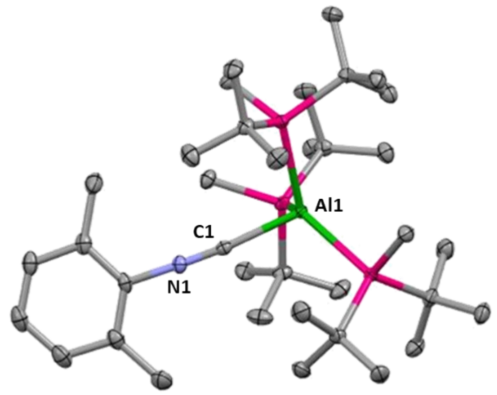

Figure S23: Molecular structure of **4**. Thermal ellipsoids are shown at a 50% probability level. H atoms are omitted for clarity. Selected bond lengths [Å] and angles [°]: C1-Al1 2.093(1), N1-C1-Al1 169.93(9).

Table S1: Crystallographic details of **4**.

**CCDC number**	2543012
**Empirical formula**	$C_{36}H_{72}AlNSi_3$
**Formula weight**	630.19
**Temperature [K]**	100
**Crystal system**	monoclinic
**Space group (number)**	$P2_1/n$ (14)
***a* [Å]**	13.0203(13)
***b* [Å]**	17.9076(16)
***c* [Å]**	17.2429(18)
**α [°]**	90
**β [°]**	95.970(4)
**γ [°]**	90
**Volume [Å^3]**	3998.6(7)
Z	4
**ρ_{calc} [gcm^{-3}]**	1.047
**μ [mm^{-1}]**	0.164
***F*(000)**	1400
**Radiation**	MoK_α (λ = 0.71073 Å)
**2θ range [°]**	3.74 to 51.38
**Index ranges**	$-15 \le h \le 15$<br>$-21 \le k \le 21$<br>$-21 \le l \le 21$

**Independent reflections**	7565
**Completeness to θ = 25.242°**	0.995
**Data / Restraints / Parameters**	7565 / 104 / 471
**Absorption correction T_{min}/T_{max} (method)**	0.720 / 0.745 (multi-scan)
**Goodness-of-fit on F^2**	1.053
**Final *R* indexes [$I \geq 2\sigma(I)$]**	R_1 = 0.0283<br>wR_2 = 0.0769

3.4 Molecular structure of **5**

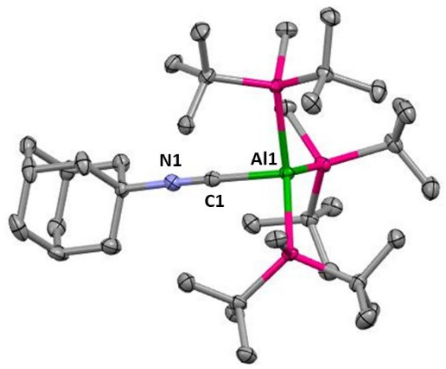

Figure S24: Molecular structure of **5**. Thermal ellipsoids are shown at a 50% probability level. H atoms are omitted for clarity. Selected bond lengths [Å] and angles [°]: C1-Al1 2.086(2), N1-C1-Al1 177.3(2).

Table S2: Crystallographic details of **5**.

**CCDC number**	2543016
**Empirical formula**	$C_{38}H_{78}AlNSi_3$
**Formula weight**	660.26
**Temperature [K]**	100
**Crystal system**	monoclinic
**Space group (number)**	$P2_1/c$ (14)
***a* [Å]**	13.7786(17)
***b* [Å]**	13.5158(17)
***c* [Å]**	22.678(3)
**α [°]**	90
**β [°]**	96.784(5)
**γ [°]**	90
**Volume [Å³]**	4193.7(9)
Z	4
**ρ_{calc} [gcm⁻³]**	1.046
**μ [mm⁻¹]**	0.159
***F*(000)**	1472
**Radiation**	MoK_α (λ=0.71073 Å)
**2θ range [°]**	4.47 to 52.91
**Index ranges**	−17 ≤ h ≤ 17<br>−16 ≤ k ≤ 16<br>−28 ≤ l ≤ 28

**Independent reflections**	8591
**Completeness to θ = 25.242°**	99.4 %
**Data / Restraints / Parameters**	8591 / 0 / 409
**Absorption correction T_{min}/T_{max} (method)**	0.680 / 0.745 (multi-scan)
**Goodness-of-fit on F^2**	1.203
**Final *R* indexes [$I \geq 2\sigma(I)$]**	R_1 = 0.0465<br>wR_2 = 0.1487

3.5 Molecular structure of **6**

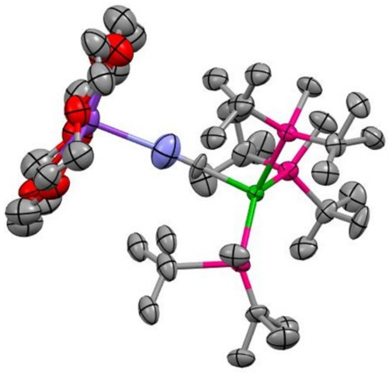

Figure S25: Connectivity of **6**.

3.6 Molecular structure of **7**

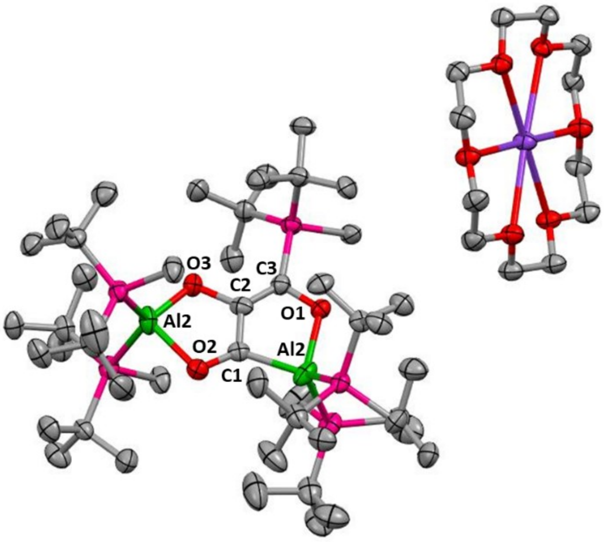

Figure S26: Molecular structure of **7**. Thermal ellipsoids are shown at a 50% probability level. H atoms are omitted for clarity. Selected bond lengths [Å] and angles [°]: Al1-O3 1.718(4), Al1-O2 1.998(4), C2-O3 1.501(5), C1-O2 1.291(7), C2-C3 1.322(5), C1-C2 1.380(7), C3-O1 1.341(6), Al2-O1 2.006(4), Al2-C1 1.954(6).

Table S3: Crystallographic details of **7**.

**CCDC number**	2543019
**Empirical formula**	$C_{68}H_{144}Al_2KN_0O_{11}Si_5$
**Formula weight**	1371.34
**Temperature [K]**	100
**Crystal system**	monoclinic
**Space group (number)**	$P2_1/n$
a **[Å]**	19.6166(11)
b **[Å]**	21.0516(12)
c **[Å]**	21.7848(12)
**α [°]**	90
**β [°]**	91.664(2)
**γ [°]**	90
**Volume [Å³]**	8992.5(9)
Z	4
**ρ_{calc} [gcm⁻³]**	1.013

**μ [mm^{-1}]**	0.191
***F*(000)**	3020
**Radiation**	MoK_α (λ=0.71073 Å)
**2θ range [°]**	3.87 to 55.14
**Index ranges**	−25 ≤ h ≤ 25<br>−27 ≤ k ≤ 27<br>−28 ≤ l ≤ 28
**Independent reflections**	20708
**Completeness to θ = 25.242°**	99.4 %
**Data / Restraints / Parameters**	20708 / 2565 / 1212
**Absorption correction T_{min}/T_{max} (method)**	0.711 / 0.746 (multi-scan)
**Goodness-of-fit on F^2**	1.189
**Final *R* indexes [$I \geq 2\sigma(I)$]**	R_1 = 0.0655<br>wR_2 = 0.2244

4 References

[1] T.J. Barton and C.R. Tully, *The Journal of Organic Chemistry* **1978**, 43, 3649-3653.

[2] N. Wiberg and C.-K. Kim, *Chem. Ber.* **1986**, 119, 2980-2994.

[3] M. Nakamoto, T. Yamasaki, and A. Sekiguchi, *J. Am. Chem. Soc.* **2005**, 127, 6954-6955.

[4] B.L.L. Réant, V.E.J. Berryman, A.R. Basford, L.E. Nodaraki, A.J. Wooles, F. Tuna, N. Kaltsoyannis, D.P. Mills, and S.T. Liddle, *J. Am. Chem. Soc.* **2021**, 143, 9813-9824.

[5] G.R. Fulmer, A.J.M. Miller, N.H. Sherden, H.E. Gottlieb, A. Nudelman, B.M. Stoltz, J.E. Bercaw, and K.I. Goldberg, *Organometallics* **2010**, 29, 2176-2179.

[6] A.S. Bruker and A. Bruker, *Acta Crystallogr., Sect. A: Found. Crystallogr* **1990**, 46, 467-473.

[7] SADABS, *SADABS*. 2016, Bruker AXS Inc.: Madison, Wisconsin, USA.

[8] *APEX suite of crystallographic software, APEX 3, version 2015.5-2*. 2015, Bruker AXS Inc.: Madison, Wisconsin, USA.

[9] G.M. Sheldrick, *Acta Crystallographica Section C: Crystal Structure Communications* **2015**, 71, 3-8.

[10] G.M. Sheldrick, *Acta Crystallographica Section A: Foundations of Crystallography* **2015**, 71, 3-8.

[11] C.B. Huebschle, G.M. Sheldrick, and B. Dittrich, *J. Appl. Cryst.* **2011**, 44, 1281.

[12] G.M. Sheldrick, *SHELXL-97*. 1998, University of Göttingen: Göttingen, Germany.

[13] A.J.C. Wilson, *International Tables for Crystallography, Vol. C* **1992**,Kluwer Academic Publishers: Dordrecht, Netherlands

[14] A. Spek, *Acta Crystallographica Section C* **2015**, 71, 9-18.

[15] A.L. Spek, *PLATON, A Multipurpose Crystallographic Tool*. 2010, Utrecht University: Utrecht, Netherlands

[16] C.F. Macrae, I. Sovago, S.J. Cottrell, P.T.A. Galek, P. McCabe, E. Pidcock, M. Platings, G.P. Shields, J.S. Stevens, M. Towler, and P.A. Wood, *J. Appl. Crystallogr.* **2020**, 53, 226-235.

[17] D. Kratzert, *FinalCif*.
